# Derivation of Multipotent Mesenchymal Precursors from Human Embryonic Stem Cells

**DOI:** 10.1371/journal.pmed.0020161

**Published:** 2005-06-28

**Authors:** Tiziano Barberi, Lucy M Willis, Nicholas D Socci, Lorenz Studer

**Affiliations:** 1Laboratory of Stem Cell and Tumor Biology, Division of Neurosurgery and Developmental Biology ProgramSloan-Kettering Institute, New York, New YorkUnited States of America; **2**Computational Biology Center, Sloan-Kettering InstituteNew York, New YorkUnited States of America; Albany Medical CollegeUnited States of America

## Abstract

**Background:**

Human embryonic stem cells provide access to the earliest stages of human development and may serve as a source of specialized cells for regenerative medicine. Thus, it becomes crucial to develop protocols for the directed differentiation of embryonic stem cells into tissue-restricted precursors.

**Methods and Findings:**

Here, we present culture conditions for the derivation of unlimited numbers of pure mesenchymal precursors from human embryonic stem cells and demonstrate multilineage differentiation into fat, cartilage, bone, and skeletal muscle cells.

**Conclusion:**

Our findings will help to elucidate the mechanism of mesoderm specification during embryonic stem cell differentiation and provide a platform to efficiently generate specialized human mesenchymal cell types for future clinical applications.

## Abstract 2

Lorenz Studer and colleagues describe the use of embryonic stem cells to derive mesenchymal precursors and then fat, cartilage, bone, and skeletal muscle cells.

## Introduction

Embryonic stem (ES) cells are pluripotent cells derived from the inner cell mass of the blastocyst that can be maintained in culture for an extended period of time without losing differentiation potential. The successful isolation of human ES cells (hESCs) has raised the hope that these cells may provide a universal tissue source to treat many human diseases. However, directed differentiation of hESCs into specific tissue types poses a formidable challenge. Protocols are currently available for only a few cell types, mostly of neural identity [1–3], and differentiation into many of the cell types derived from the paraxial mesoderm has not been reported, with the exception of a recent study indicating osteoblastic differentiation [4]. Mesenchymal stem cells (MSCs) have been isolated from the adult bone marrow [5], adipose tissue [6], and dermis and other connective tissues [7]. Harvesting MSCs from any of these sources requires invasive procedures and the availability of a suitable donor. The number of MSCs that can be obtained from a single donor is limited, and the capacity of these cells for long-term proliferation is rather poor. In contrast, hESCs could provide an unlimited number of specialized cells. In this study, we present techniques for the generation and purification of mesenchymal precursors from hESCs and their directed differentiation in vitro into various mesenchymal derivatives, including skeletal myoblasts. Our isolation method for mesenchymal precursors is the first example, to our knowledge, of efficiently deriving structures of the paraxial mesoderm from ES cells, and further highlights the potential of hESCs for basic biology and regenerative medicine.

## Methods

### Cell Culture and FACS

Undifferentiated hESCs, H1 (WA-01, XY, passages 40–65) and H9 (WA-09, XX, passages 35–45), were cultured on mitotically inactivated mouse embryonic fibroblasts (Specialty Media, Phillipsburg, New Jersey, United States) and maintained under growth conditions and passaging techniques described previously [3]. OP9 cells were maintained in alpha MEM medium containing 20% fetal bovine serum (FBS) and 2 mM L-glutamine. Mesenchymal differentiation was induced by plating 10 × 10^3^ to 25 × 10^3^ cells/cm^2^ on a monolayer of OP9 cells in the presence of 20% heat-inactivated FBS in alpha MEM medium. Flow-activated cell sorting (FACS) (CD73-PE; PharMingen, San Diego, California, United States) was performed on a MoFlo (Cytomation, Fort Collins, Colorado, United States). All human ES cell–derived mesenchymal precursor cell (hESMPC) lines in this study are of polyclonal origin. Primary human bone marrow–derived MSCs and primary human foreskin fibroblasts (both from Poietics, Cambrex, East Rutherford, New Jersey, United States) were grown in alpha MEM medium containing 10% FBS and 2 mM L-glutamine.

### Adipocytic Differentiation

hESMPCs are grown to confluence followed by exposure to 1 mM dexamethasone, 10 μg/ml insulin, and 0.5 mM isobutylxanthine (all from Sigma, St. Louis, Missouri, United States) in alpha MEM medium containing 10% FBS for 2–4 wk. Data were confirmed in hESMPC-H1.1, -H1.2, -H1.3, and -H9.1 (hESMPC-H1.4 was not tested).

### Chondrocytic Differentiation

Differentiation of hESMPCs was induced in pellet culture [5] by exposure to 10 ng/ml TGF-β3 (R & D Systems, Minneapolis, Minnesota, United States) and 200 μM ascorbic acid (Sigma) in alpha MEM medium containing 10% FBS for 3–4 wk. Data were confirmed in hESMPC-H1.1, -H1.3, and -H9.1 (hESMPC-H1.2 and -H1.4 were not tested).

### Osteogenic Differentiation

hESMPCs were plated at low density (1 × 10^3^ to 2.5 × 10^3^ cells/cm^2^) on tissue-culture-treated dishes in the presence of 10 mM β-glycerol phosphate (Sigma), 0.1 μM dexamethasone, and 200 μM ascorbic acid in alpha MEM medium containing 10% FBS for 3–4 wk. Data were confirmed in hESMPC-H1.1, -H1.3, and -H9.1 (hESMPC-H1.2 and -H1.4 were not tested).

### Myogenic Differentiation

Confluent hESMPCs were maintained for 2–3 wk in alpha MEM medium with 20% heat-inactivated FBS. More rapid induction was observed in the presence of medium conditioned for 24 h by differentiated C2C12 cells. Coculture of hESMPCs and C2C12 cells was carried out in alpha MEM with 3% horse serum and 1% FBS [8]. Data were confirmed in hESMPC-H1.3, -H1.4, and -H9.1 (hESMPC-H1.1 and -H1.2 were not tested).

### Cytochemistry

Immunocytochemistry for all surface markers was performed on live cells. Monoclonal antibodies VCAM, STRO-1, ICAM-1(CD54), CD105, CD29, and MF20 were from Developmental Studies Hybridoma Bank (University of Iowa, Iowa City, Iowa, United States); CD73, CD44, and ALCAM(CD166) were from BD Biosciences Pharmingen (San Diego, California, United States). All other immunocytochemical analyses were performed after fixation in 4% paraformaldehyde and 0.15% picric acid, followed by permeabilization in 0.3% Triton X100. Polyclonal antibodies used were MyoD (Santa Cruz Biotechnology, Santa Cruz, California, United States) and nestin (gift from R. McKay); monoclonal antibodies were vimentin, alpha smooth muscle actin, fast-switch myosin, pan-cytokeratin (all from Sigma), and human nuclear antigen (Chemicon, Temecula, California, United States).

Alkaline phosphatase reaction was performed using a commercially available kit (Kit-86; Sigma) and the mineral was stained with silver nitrate according to the von Kossa method. Fat granules were visualized by Oil Red O staining solution (Sigma). Alcian Blue (Sigma) was used to detect extracellular matrix proteoglycans in chondrogenic cultures.

### Gene-Expression Analyses

#### RT-PCR analysis

Total RNA was extracted by using the RNeasy kit and DNase I treatment (Qiagen, Valencia, California, United States). Total RNA (2 μg each) was reverse transcribed (SuperScript; Invitrogen, Carlsbad, California, United States). PCR conditions were optimized and linear amplification range was determined for each primer by varying annealing temperature and cycle number. PCR products were identified by size, and identity was confirmed by DNA sequencing. Primer sequences, cycle numbers, and annealing temperatures are provided in Table S1.

#### Affymetrix analysis

Total RNA (5 μg) from primary MSCs, from hESMPC-H9.1, hESMPC-H1.2, and three samples of undifferentiated hESCs (H1; passages 42–46), were processed by the Memorial Sloan-Kettering Cancer Center Genomics Core Facility and hybridized on Affymetrix (Santa Clara, California, United States) U133A human oligonucleotide arrays. Data were analyzed using MAS5.0 (Affymetrix) software. Transcripts selectively expressed in each of the mesenchymal cell populations (MSC, hESMPC-H9.1, and hESMPC-H1.2) were defined as those called “increased” by the MAS5.0 algorithm in each of three comparisons with independent samples of undifferentiated hESCs. A Venn diagram was generated to visualize overlap in gene expression. Further statistical analyses were performed as described below.

## Results

**Figure 1:**
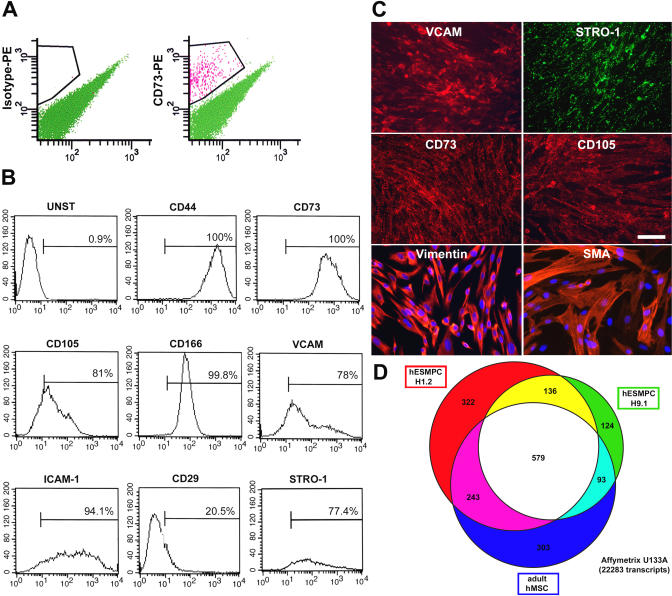
Isolation and Characterization of hESMPCs (A) FACS (MoFlo, Cytomation) for the isolation of CD73+ precursors (right) and isotype control (left). (B) Flow cytometry analysis of the CD73+ hESMPC population for various markers characteristic of MSCs, including CD44, CD73, CD105, CD166, VCAM, ICAM-1, CD29, and STRO-1. (C) Immunocytochemistry of hESMPCs for MSC markers (VCAM, STRO-1, CD73, and CD105). The cells also express vimentin and alpha smooth muscle actin. Scale bar = 50 μm. (D) Venn diagram presenting the overlap among transcripts selectively expressed in hESMPC-H1.2, hESMPC-H9.1, and primary adult human MSCs.

Mesenchymal differentiation of hESCs (lines H1 [WA-01] and H9 [WA-09]) [9] was induced by plating undifferentiated hESCs on a monolayer of murine OP9 stromal cells [10], in the presence of 20% heat-inactivated FBS in alpha MEM medium. OP9 cells have been previously shown to induce blood cell differentiation from mouse ES cells [11]. After 40 d of coculture, cells were harvested and sorted by FACS for CD73, a surface marker expressed in adult MSCs [5] (Figure 1A). An average of 5% CD73+ cells was obtained from the mixed culture of OP9 and differentiated hESC progeny. CD73+ cells were replated in the absence of stromal feeders on tissue culture plates and expanded in alpha MEM medium with 20% FBS for 7–14 d. We next established the membrane antigen profile of the resulting population of flat spindle-like cells. The H1- and H9-derived CD73+ cells expressed a comprehensive set of markers that are considered to define adult MSCs, including CD105(SH2), STRO-1, VCAM (CD106), CD29(integrin β1), CD44, ICAM -1(CD54), ALCAM(CD166), vimentin, and alpha smooth muscle actin (Figure 1B and 1C). The cells were negative for hematopoietic markers such as CD34, CD45, and CD14. They were also negative for neuroectodermal, epithelial, and muscle cell markers including nestin, pancytokeratin, and desmin (data not shown). The human identity of these presumed mesenchymal cells (termed hESMPC-H1.1, -H1.2, -H1.3, -H1.4, and -H9.1) was confirmed for all experiments by immunocytochemistry for human nuclear antigen to rule out the possibility of contamination with OP9 cells (Figure S1).

To further characterize hESMPCs, we performed genome-wide expression analysis using oligonucleotide arrays (Affymetrix U133A). The expression profiles of hESMPC-H1.2 and hESMPC-H9.1 were compared with that of human primary adult MSCs. Housekeeping genes for each of the mesenchymal cell populations were eliminated by subtracting those transcripts also expressed in at least one of three independent samples of undifferentiated hESCs. Based on this analysis, 1,280 transcripts were selectively expressed in hESMPC-H1.2, 932 transcripts in hESMPC-H9.1, and 1,218 transcripts in primary adult MSCs. A remarkable overlap of 579 transcripts shared among the three mesenchymal populations was observed (Figure 1D). Using the genes that were selected in the initial filter, we performed a statistical analysis on the expression levels to determine whether the genes were expressed significantly differently in the two cell types. We used a Bayesian extension to the standard *t*-test [12] to assess this difference. Of the 579 genes, 412 of them were significantly different, at a false discovery rate cutoff of 0.05. The relative fold changes were also extremely large in many of the cases. We also looked at the variance of the expression levels within the cell types. For the MSCs, 94% had a coefficient of variation less than 20% for the expression (log transformed); for the ES-derived cells, 72% had a coefficient of variation less than 20%. Numerous known MSC markers were included in the list of 412 genes, such as the *mesenchymal stem cell protein* DSC54 (13.9-fold increase, *p* < 0.001), *neuropilin 1* (30.4-fold increase, *p* < 0.001), *hepatocyte growth factor* (48.1-fold increase, *p* < 0.001), *forkhead box D1* (14.8-fold increase, *p* < 0.001), and *notch homolog 2* (2.9-fold increase, *p* < 0.001) . Table S2 lists the *p*-values from the test, the mean and standard deviation of the expression levels, and the relative fold change of all 412 genes between the two types.

Known markers of MSCs, such as mesenchymal stem cell protein DSC54, were all included within the 579 shared transcripts. These findings support the immunocytochemical data and suggest that hESMPCs and primary MSCs are highly related.

**Figure 2:**
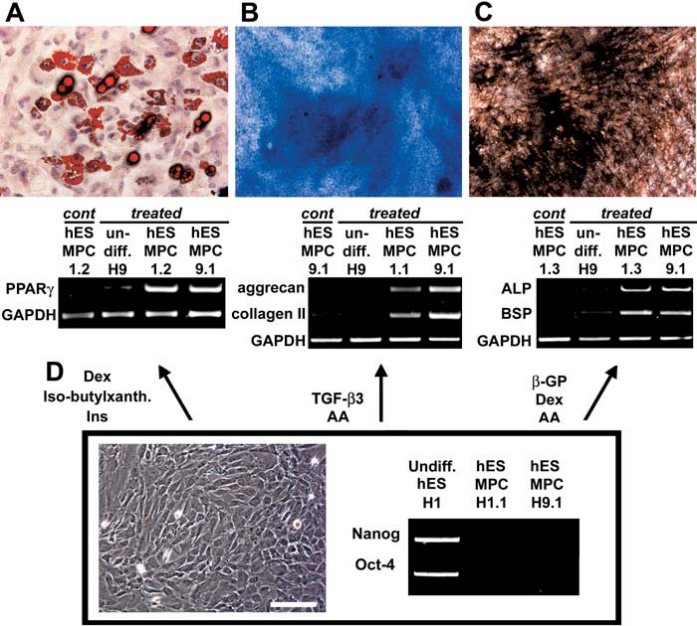
Selective Differentiation of hESMPCs into Various Mesenchymal Derivatives (A) Adipocytic differentiation in the presence of dexamethasone, insulin, and isobutylxanthine. Adipocytic characterization by Oil Red O staining and RT-PCR analysis for PPARγ. (B) Chondrocytic differentiation in the presence of TGF-β3 and ascorbic acid. Chondrocytic characterization by Alcian Blue staining and RT-PCR for aggrecan and collagen II. (C) Osteogenic differentiation in the presence of β-glycerolphosphate, dexamethasone, and ascorbic acid. Osteocytic characterization by von Kossa staining and RT-PCR for bone-specific alkaline phosphatase (ALP) and bone sialoprotein (BSP). (D) Phase-contrast image of hESMPCs and RT-PCR for the ES cell markers *Nanog* and *Oct-4 i*n hESMPC-H1.1 and -H9.1 compared with undifferentiated H1 hESCs. Scale bar = 50 μm for all panels.

MSCs are characterized functionally by their ability to differentiate into mesenchymal tissues, such as fat, cartilage, and bone. Therefore, we tested whether hESMPCs have the same potential (Figure 2).

Adipocytic differentiation of hESMPCs was induced under conditions described previously for primary adult MSCs [5]. Appearance of cells harboring fat granules was observed after 10–14 d in culture. After 3 wk of induction, more than 70% of the cells displayed Oil Red O+ fat granules, and *PPARγ,* a marker of adipocytic differentiation, was detected by RT-PCR. (Figure 2A).

Chondrocytic differentiation was achieved using the pellet culture system [5]. After 28 d in culture, more than 50% of all cells exhibited robust staining for Alcian Blue, a marker specific for extracellular matrix proteoglycans. Chondrocytic differentiation was confirmed by the gene expression of *collagen II* and *aggrecan*, two components of extracellular matrix selectively expressed by chondrocytes, using RT-PCR (Figure 2B).

Osteogenic differentiation was induced in the presence of β-glycerolphosphate [5]. Osteogenesis was demonstrated by specific staining for calcium deposition in the matrix (von Kossa, Figure 2C; or Alizarin Red, Figure S2A) and increased expression of *bone-specific alkaline phosphatase* and *bone sialoprotein* at day 28 of treatment (Figures 2C and S2B). At day 28, Alizarin Red staining was detected in approximately 70% of all cells. Throughout these studies, human adult MSCs and foreskin fibroblasts were used as positive and negative controls, respectively.

**Figure 3:**
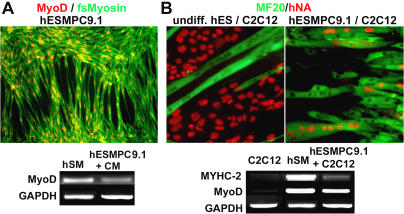
Myogenic Differentiation of hESMPCs (A) Immunocytochemistry for MyoD (red) and fast-switch myosin (green). RT-PCR for *MyoD i*n human skeletal muscle as a positive control (hSM), and in hESMPC-H9.1 cells differentiated for 10 d in the presence of C2C12-conditioned medium (hESMPC). (B) Myotube formation induced at high cell densities in the presence of C2C12 cells. Myotube characterization by immunocytochemistry for MF20 against sarcomeric myosin (green) and human nuclear antigen (hNA, red). Left panel: Control undifferentiated hESCs (H9) do not fuse with C2C12. Right panel: Under identical culture conditions, hESMPCs (line 9.1) efficiently fuse with C2C12 cells, forming myotubes containing human nuclei. RT-PCR for human specific muscle transcripts *myosin heavy chain IIa (MYHC-2)* and *MyoD* in C2C12 cells, in human skeletal muscle as positive control (huSM), and in hESMPC-H9.1 cells cocultured with C2C12 cells.

In addition to adipocytic, chondrocytic, and osteogenic differentiation, reports suggested that adult MSCs can form skeletal muscle [13]. Although generation of skeletal muscle cells from adult MSCs remains controversial, we tested whether hESMPCs exhibit this potential. Under the conditions previously described [13], hESMPC-H1.1 and -H9.1 did not yield significant numbers of MyoD+ cells after 15–20 d in culture. However, when confluent cells were maintained in culture in the presence or absence of 5-AzaC without passage for more than 21 d, expression of specific skeletal muscle markers such as MyoD and fast-switch myosin was observed (Figure 3A). More rapid myogenic differentiation was obtained in the presence of 24-h-conditioned medium from the murine myoblastic cell line C2C12 previously induced to form myotubes [14]. Direct coculture of hESMPCs with C2C12 cells led to the formation of hESMPC-derived myotubes, as visualized by expression of human nuclear antigen (Figure 3B), similar to those formed by host C2C12 cells. After 1 wk of coculture, myotubes composed of human nuclei accounted for more than 10% of the total number of human cells present, and each human myotube was composed of up to ten human nuclei. Human cell contribution to myotubes in coculture was confirmed by expression of human muscle-specific transcripts such as *MyoD, myosin heavy chain IIa*, and *myogenin* (data not shown). These data demonstrate that hESMPCs can give rise to mesenchymal derivatives typically obtained from primary adult MSCs, as well as to cells expressing markers of skeletal muscle.

One concern for the clinical application of hESC-derived progeny in regenerative medicine is the risk of teratoma formation due to the presence of residual undifferentiated ES cells among the differentiated progeny. We did not detect markers of undifferentiated hESCs, such as *Nanog* [15] or *Oct-4* [16], in any of the hESMPCs by RT-PCR (see Figure 2D) and immunocytochemistry (data not shown), suggesting the lack of any undifferentiated ES cells in hESMPC cultures. However, future in vivo studies are required to rule out the potential of these cells for teratoma formation.

## Discussion

Previous studies have demonstrated the derivation of neural cells [1–3], hematopoietic [17] and endothelial lineages [18], and cardiomyocytes [19] from hESCs. This study presents the induction of paraxial mesoderm with the generation of multipotent mesenchymal precursors. We calculate that under these conditions a single undifferentiated hESC yields an average of one CD73+ cell at day 40 of differentiation, suggesting a balance between cell proliferation and cell selection. There were no obvious differences in marker and gene-expression profile or in differentiation behavior among the five hESMPC lines generated. However, some of the lines (e.g., hESMPC9.1) exhibited a tendency of spontaneous osteogenic differentiation after long-term propagation. Directed differentiation of hESCs into somatic stem-cell-like precursors represents a substantial advancement in harnessing the developmental potential of hESCs. The high purity, unlimited availability, and multipotentiality of hESMPCs will provide the basis for future therapeutic efforts using these cells in preclinical animal models of disease. Such in vivo studies will also be required to properly assess the safety profile of these cells. Furthermore, our system also offers a novel platform to study basic mechanisms of mesodermal induction and differentiation during early human development.

## Supporting Information

Figure S1 Human Identity of CD73+ Cells after FACS All cells as visualized by DAPI+ nuclei express human nuclear antigen (hNA) confirming the absence of any contaminating OP9 cells. Scale bar = 50 μm.
(148 KB PDF).

Figure S2 Additional Markers of Bone Differentiation (A) Alizarin Red staining for calcium deposition in the matrix in hESMPCs untreated (left panel) or treated in the presence of β-glycerolphosphate (right panel; compare to Figure 2C).
(B) Increasing alkaline phosphatase reactivity during osteogenic differentiation of hESMPC-H1.1. Scale bar = 250 μm for main panels, 50 μm for insets.
(278 KB PDF).

Table S1 All Primers Used in This Study (22 KB PDF).

Table S2 List of Shared Genes List of 421 genes that are shared between primary and hESC-derived mesenchymal precursors but significantly different from undifferentiated hESCs (see main text for details).
(107 KB XLS).

### Accession Numbers

The Gene Expression Omnibus (GEO) (http://www.ncbi.nlm.nih.gov/geo) accession number for all raw microarray data used in this study is GSE2248.

The Unigene (http://www.ncbi.nlm.nih.gov/entrez/query.fcgi?db=unigene) accession numbers for the gene products discussed in this paper are aggrecan (Hs.2159 [http://www.ncbi.nlm.nih.gov/UniGene/clust.cgi?ORG=Hs&CID=2159; bone sialoprotein (Hs.518726 [http://www.ncbi.nlm.nih.gov/UniGene/clust.cgi?ORG=Hs&CID=518726; bone-specific alkaline phosphatase (Hs.75431 [http://www.ncbi.nlm.nih.gov/UniGene/clust.cgi?ORG=Hs&CID=75431; collagen II (Hs.408182 [http://www.ncbi.nlm.nih.gov/UniGene/clust.cgi?ORG=Hs&CID=408182; forkhead box D1 (Hs.519385 [http://www.ncbi.nlm.nih.gov/UniGene/clust.cgi?ORG=Hs&CID=519385; hepatocyte growth factor (Hs.396530 [http://www.ncbi.nlm.nih.gov/UniGene/clust.cgi?ORG=Hs&CID=396530; mesenchymal stem cell protein (DSC54, Hs.157461 [http://www.ncbi.nlm.nih.gov/UniGene/clust.cgi?ORG=Hs&CID=157461; MyoD (Hs.520119 [http://www.ncbi.nlm.nih.gov/UniGene/clust.cgi?ORG=Hs&CID=520119; myogenin (Hs.2830 [http://www.ncbi.nlm.nih.gov/UniGene/clust.cgi?ORG=Hs&CID=2830; myosin heavy chain IIa (Hs.513941 [http://www.ncbi.nlm.nih.gov/UniGene/clust.cgi?ORG=Hs&CID=513941; Nanog (Hs.329296 [http://www.ncbi.nlm.nih.gov/UniGene/clust.cgi?ORG=Hs&CID=329296]) [15]; neuropilin 1 (Hs.131704 [http://www.ncbi.nlm.nih.gov/UniGene/clust.cgi?ORG=Hs&CID=131704; notch homolog 2 (Hs.549056 [http://www.ncbi.nlm.nih.gov/UniGene/clust.cgi?ORG=Hs&CID=549056; Oct-4 (Hs.504658 [http://www.ncbi.nlm.nih.gov/UniGene/clust.cgi?ORG=Hs&CID=504658; and PPARγ (Hs.162646 [http://www.ncbi.nlm.nih.gov/UniGene/clust.cgi?ORG=Hs&CID=162646]).

**Patient Summary**

**Background**

The discovery and isolation of human embryonic stem cells (cells that are capable of renewing themselves and turning into the many different cell types that make up the human body) has the potential to revolutionize the treatment of many diseases that require the replacement of abnormal or missing cells. In particular, it would be very valuable to be able to replace tissues that are derived from one particular tissue type—mesenchyme—which bone, cartilage, fat and muscle develop from. However, before such treatments can happen, it will be necessary to work out exactly how embryonic cells become other cells, and whether it is possible to make these changes happen in the laboratory.

**What Did the Researchers Do?**

They took two lines of completely undifferentiated human embryonic stem cells and by culturing them in the presence of mouse cells stimulated them to turn into mesenchymal cells. They then treated these cells with compounds to make them change into specialized bone, cartilage, fat, and muscle cells. They were able to confirm that these cells were all human (important because the early part of the experiment is done in the presence of mouse cells) and also that there was no evidence that the cells became cancerous.

**What Do These Findings Mean?**

It is theoretically possible to produce lines of bone, cartilage, fat, and muscle cells from human embryonic stem cells. However, the process will need more refinement before the cell lines could be used for treatment; ideally, for example, all the culturing would be done without any mouse cells.

**Where Can I Get More Information?**

The United States National Institutes of Health has a group of Web pages on stem cells: http://stemcells.nih.gov/info/faqs.asp

The International Society for Stem Cell Research has a list of frequently asked questions about stem cells: http://www.isscr.org/science/faq.htm

## Abbreviations

ES: embryonic stem
FACS: flow-activated cell sorting
FBS: fetal bovine serum
hESC: human embryonic stem cell
hESMPC: human embryonic stem cell–derived mesenchymal precursor cell
MSC: mesenchymal stem cell
