# Supplementary figures and images for: Derivation of Multipotent Mesenchymal Precursors from Human Embryonic Stem Cells
Source: PLoS Med. 2005 Jun 28;2(6):e161. doi: 10.1371/journal.pmed.0020161 (PMC1160574; doi:10.1371/journal.pmed.0020161)

**DAPI**

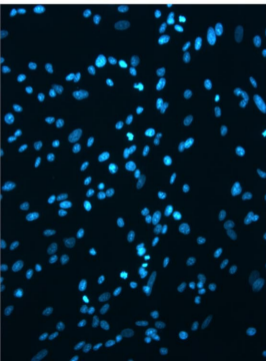

**hNA**

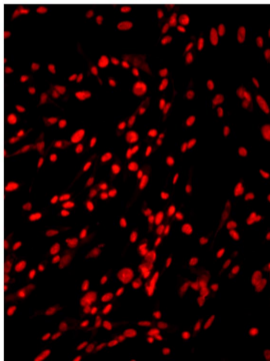

**DAPI/hNA**

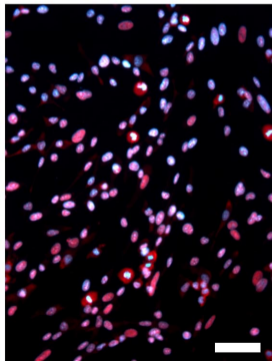

Supplement: Figure S1 — All cells as visualized by DAPI+ nuclei express human nuclear antigen (hNA) confirming the absence of any contaminating OP9 cells. Scale bar = 50 μm. (148 KB PDF). [file pmed.0020161.sg001.pdf]

**A**      **untreated**

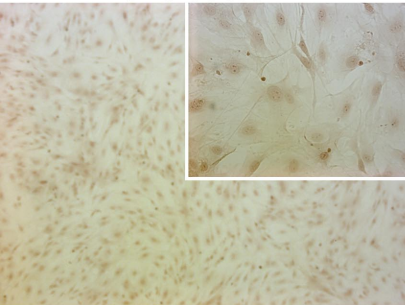

**treated**

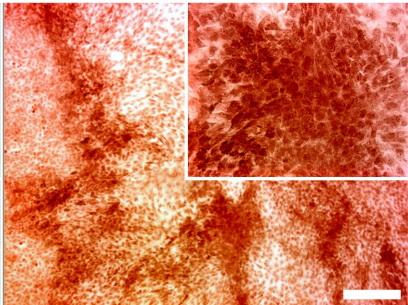

**B**      **day0**

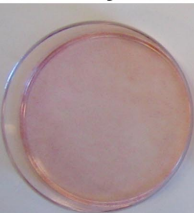

**day7**

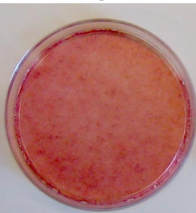

**day14**

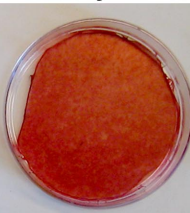

**day19**

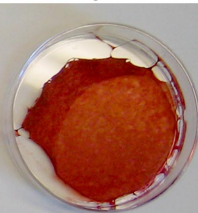

Supplement: Figure S2 — (A) Alizarin Red staining for calcium deposition in the matrix in hESMPCs untreated (left panel) or treated in the presence of β-glycerolphosphate (right panel; compare to Figure 2C). (B) Increasing alkaline phosphatase reactivity during osteogenic differentiation of hESMPC-H1.1. Scale bar = 250 μm for main panels, 50 μm for insets. (278 KB PDF). [file pmed.0020161.sg002.pdf]
