# Supplementary material for: Derivation of Multipotent Mesenchymal Precursors from Human Embryonic Stem Cells
Source: PLoS Med. 2005 Jun 28;2(6):e161. doi: 10.1371/journal.pmed.0020161 (PMC1160574; doi:10.1371/journal.pmed.0020161)
Supplement: Table S1 — (22 KB PDF). [file pmed.0020161.st001.pdf]

Barberi et al., Supplementary Table 1

Name	orientation	Annealing temp.	Size of amplified fragment	Sequence
GAPDH	sense	59	342	*CCCCTTCATTGACCTCAACTACA*
	antisense	59		*TTGCTGATGATCTTGAGGCTGT*
OCT4	sense	59	422	*ATTTGCCAAGCTCCTGAAGCAG*
	antisense	59		*TTGATCGCTTGCCCTTCTGG*
NANOG	sense	56	378	*AGTGTGGATCCAGCTTGTCCC*
	antisense	56		*TTCTTGCATCTGCTGGAGGC*
PPARγ	sense	56	350	*CTCCTATTGACCCAGAAAGC*
	antisense	56		*GTAGAGCTGAGTCTTCTCAG*
COLLAGEN 2	sense	58	390	*ATGATTCGCCTCGGGGCTCC*
	antisense	58		*CATTACTGGGAACTGGGCGC*
AGGRECAN	sense	56	190	*CACTGTTACCGCCACTTCCC*
	antisense	56		*ACCAGCGGAAGTCCCCTTCG*
ALP	sense	51	454	*TGGAGCTTCAGAAGCTCAACACCA*
	antisense	51		*ATCTCGTTGTCTGAGTACCAGTCC*
BSP	sense	56	450	*AATGAAAACGAAGAAAGCGAAG*
	antisense	56		*ATCATAGCCATCGTAGCCTTGT*
MyoD	sense	55	170	*GCAGGTGTAACCGTAACC*
	antisense	55		*ACGTACAAATTCCCTGTAGC*
MYOGENIN	sense	55	438	*TAAGGTGTGTAAGAGGAAGTCG*
	antisense	55		*CCACAGACACATCTTCCACTGT*
MYH-2	sense	52	850	*CTGCTGAAGGAGAGGGAGCT*
	antisense	52		*TGATTAGCTGGTCACACCTT*
